# Smartphone Spectrometers

**DOI:** 10.3390/s18010223

**Published:** 2018-01-14

**Authors:** Andrew J. S. McGonigle, Thomas C. Wilkes, Tom D. Pering, Jon R. Willmott, Joseph M. Cook, Forrest M. Mims, Alfio V. Parisi

**Affiliations:** 1 Department of Geography, University of Sheffield, Sheffield S10 2TN, UK; tcwilkes1@sheffield.ac.uk (T.C.W.); t.pering@sheffield.ac.uk (T.D.P.); joe.cook@sheffield.ac.uk (J.M.C.); 2 School of Geosciences, The University of Sydney, Sydney 2006, Australia; 3 Department of Electronic and Electrical Engineering, University of Sheffield, Sheffield S1 4DE, UK; j.r.willmott@sheffield.ac.uk; 4 Geronimo Creek Observatory, Seguin, TX 78155, USA; fmimsiii@yahoo.com; 5 Faculty of Health, Engineering and Sciences, University of Southern Queensland, Toowoomba, QLD 4350, Australia; alfio.parisi@usq.edu.au

**Keywords:** smartphone spectrometers, smartphone spectroscopy, low cost scientific instrumentation, medical diagnostics, food quality inspection, environmental monitoring

## Abstract

Smartphones are playing an increasing role in the sciences, owing to the ubiquitous proliferation of these devices, their relatively low cost, increasing processing power and their suitability for integrated data acquisition and processing in a ‘lab in a phone’ capacity. There is furthermore the potential to deploy these units as nodes within Internet of Things architectures, enabling massive networked data capture. Hitherto, considerable attention has been focused on imaging applications of these devices. However, within just the last few years, another possibility has emerged: to use smartphones as a means of capturing spectra, mostly by coupling various classes of fore-optics to these units with data capture achieved using the smartphone camera. These highly novel approaches have the potential to become widely adopted across a broad range of scientific e.g., biomedical, chemical and agricultural application areas. In this review, we detail the exciting recent development of smartphone spectrometer hardware, in addition to covering applications to which these units have been deployed, hitherto. The paper also points forward to the potentially highly influential impacts that such units could have on the sciences in the coming decades.

## 1. Introduction

Just as the personal computer has revolutionized research in the sciences over recent decades, smartphones are now finding increasing application in a wide variety of research domains. This is expedited by the integrated data capture and processing capability (e.g., lab in a phone) operability of these units, their compact form factor, relatively low cost, and very widespread circulation. Indeed, whilst smartphones can be interfaced with external hardware for data acquisition, they are also host to an increasingly sophisticated array of onboard sensors, e.g., proximity sensors, accelerometers, moisture sensors, gyroscopes and electro-magnetic compasses, in addition to the sensors associated with the touchscreen and the cameras themselves. Furthermore, in view of their intrinsic networking capacity, smartphones are also obvious candidates for deployment as nodes within Internet of Things networks, to enable capture of spatially resolved big data within a variety of environments.

**Figure 1:**
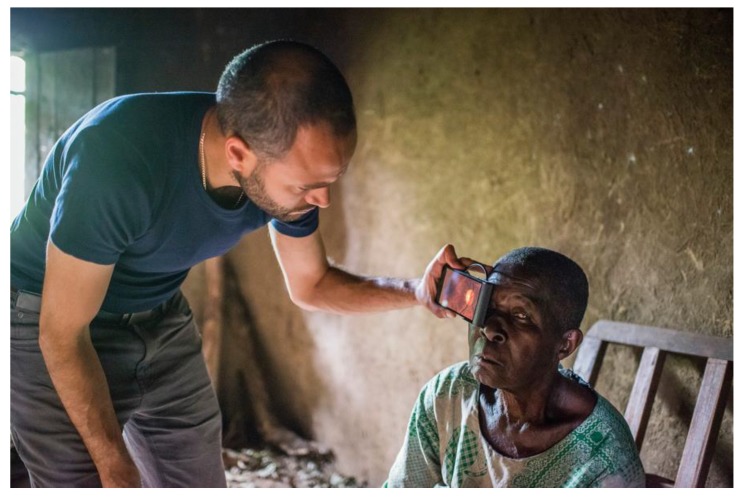
Smartphone based eye examination of a blind woman in Kenya. This work is being performed by the PEEK Vision Foundation [3,4]. ©Rolex/Joan Bardeletti.

Over the last decade smartphone imaging has been increasingly applied in a variety of scientific domains. For example, in an early seminal work on this theme, a smartphone-based microscope assembly was reported and used to identify *P. falciparum*-infected and sickle red blood cells and *M. tuberculosis*-infected sputum samples, via a combination of bright-field and fluorescence approaches [1]. The point was made, that owing to the wide spread use of smartphone technology and the availability of cell phone networks in many parts of the developing world, that this approach could be a highly cost-effective means of diagnosis and screening concerning hematologic and infectious diseases, in contexts where standard microscopy laboratory equipment is scarce. The authors go on to anticipate telemedicine for global healthcare, mediated via mobile phones. A more recent review of the field has tracked how the Moore’s law type, doubling every two years or so, of the Megapixel count of phone cameras, has expedited the uptake of smartphone imaging in microscopy (fluorescence, dark-field and bright-field), enabling imaging and detection of individual viruses and single DNA molecules [2]. In terms of point of care applications, eye health has featured too, in particular involving an adapter attached to a smartphone camera, to enable imaging of the optic nerve and retina, which is of importance in the diagnosis and monitoring of a variety of conditions including glaucoma, macular degeneration, hypertension and malaria. In an exciting recent development, this technology has been applied in a Kenyan context, with a view to expanding the reach of eye care in the developing world [3,4] (Figure 1).

The individual colour (RGB) outputs of smartphones have also been exploited in an imaging capacity in a number of ways, e.g., colourimetric determination of water quality. For instance, chlorine contamination has been characterised via reaction of this pollutant with an indicator chemical, prompting a change in colouration, which is captured by the smartphone [5]. This capacity, combined with the GPS functionality of the phone, can be used to obtain spatio-temporal maps of species concentrations [6], which would be very useful, for instance in monitoring dispersal of effluent following pollutant events. Smartphone-based colourimetry has also been realised in bioanalytical [7] applications as well as for monitoring air quality [8]. This capacity, has in addition, been applied to characterizing the colour of soils, in order to achieve objective Munsell type soil classifications [9]. Smartphone imaging has also been applied in fluorescence measurements in the visible [10,11], particularly in terms of point of care diagnostics [12], and in the infrared [13], as well as in flame emission diagnostics [14], in profiling laser beams [15] and for characterizing food volumes via an image based segmentation algorithm as a potential aid in diet monitoring [16].

Smartphone imaging has furthermore been useful in remote sensing, for example in aerial photography and grass roots mapping applications. This methodology has been applied from drones and kites [17], using the positional information available from the smartphone, along with open source geospatial toolkits, to deliver products, which can be readily fed into geographical information systems, with potential application in a variety of areas e.g., in providing rapid data to inform responses to humanitarian or natural disasters. Smartphones have additionally been applied quantitatively in this context, for example in determining ‘leaf area index’, which is a measure of foliage cover [18], and these units could be powerful tools in tracking longer term trends in sky [19], land cover and vegetation conditions. Ultraviolet characterisation with these devices has been performed too, with a view to potential future use in determining personal UV exposure levels [20,21,22,23]. Finally, there has also been considerable use of array sensors, developed for the smartphone market, but instead housed within camera modules, which are interfaced with low cost computer, e.g., Raspberry Pi, boards, for application in a number of scientific domains, e.g., volcanology [24,25]. This alternate configuration has the advantage of a potentially greater degree of user control of the sensor than available via the phone itself, e.g., by using the Python programming language.

Building on the initial application of smartphone sensor arrays in imaging, a number of workers have begun to pioneer methods of using these sensors to capture spectral information, e.g., by dispersing spectra across one dimension of the sensors’ 2D arrays. This interest has spanned the maker, academic research and citizen science communities, expedited by the advent of 3D printing, which has been used to great effect to generate open source optical hardware at a fraction of the cost of commercially available units, therefore opening optical science to everyone [26].

**Figure 2:**
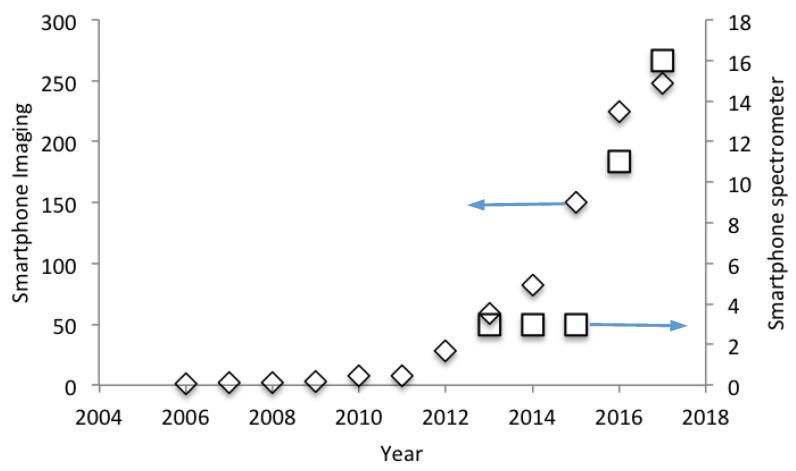
The rapid recent escalation in interest in smartphone spectrometers is evidenced by an upturn in articles on this topic in the journal literature. For reference a similar uptake in attention is apparent in respect of the earlier adoption of smartphone imaging. Please see main text for further detail.

This article provides a review of the exciting and potentially highly disruptive advent of spectral acquisition with smartphones. Such a piece is timely, as this technology is now beginning to gain traction within the sciences as units are being developed which are of sufficient quality to be useful in a wide variety of applications, including agriculture, food inspection, atmospheric spectroscopy and analytic chemistry. Indeed, Figure 2 reveals the sharp uptake in interest in this field since 2014, as manifested as research articles per year within the Science Citation Index Expanded (Clivariate Analytics; data accessed November 2017), using the search term: ‘smartphone and spectrometer’. For reference, Figure 2 also details the earlier escalation in interest in smartphone imaging, via a ‘smartphone and imaging’ search, in line with the now widespread reach of that approach within the sciences.

## 2. Transmission Grating Configurations

**Figure 3:**
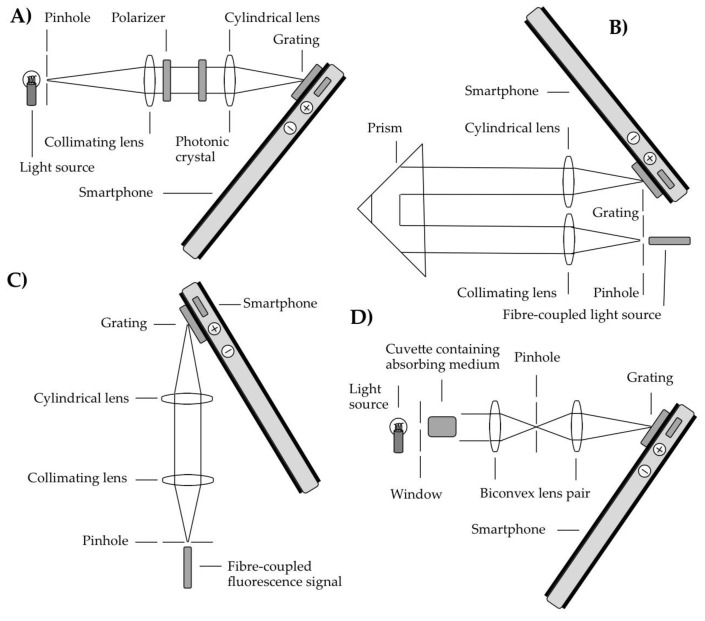
Sample schematics showing the generic operating principles of some transmissive grating smartphone spectrometer designs: (**A**) a configuration involving a pinhole, spherical then cylindrical lens [30,31], in this case shown with a photonic crystal resonant reflection assembly placed in the optical path [30]; in (**B**) a similar approach is adopted, but with evanescent wave absorption measured in the medium surrounding the prism [31]; (**C**) shows a system for measurement of fluorescence spectra, transmitted to the spectrometer, via an optical fibre [33]; in (**D**) absorbance is monitored within a cuvette, with two spherical lenses and a pinhole used to filtered out scattered light and couple radiation to the spectrometer; in this case a DVD section was also used as the diffraction grating [34]. Please see the main text for further detail.

Smartphones have been used to acquire spectra via two dominant approaches, namely using transmissive, and reflective diffraction gratings, respectively to disperse incoming radiation, before detection using the smartphone camera sensor. These modalities will be covered in this and the next section, respectively, with other formats used to acquire spectra with smartphones treated in the discussion section. In terms of transmission grating approaches, some of the case study optical configurations are shown in Figure 3.

The initial designs of smartphone spectrometers were focused on affixing transmissive diffraction gratings directly to the windows of smartphone cameras. In, what to our knowledge is the first report of a smartphone spectrometer, a system is described in which a slit shaped aperture was formed over the grating, using two parallel strips of tape, separated by ≈1 mm [27]. A tube containing darkened foil was mounted over the camera, at an angular inclination of around 45° in order for the sensor to be illuminated by the first order diffracted output from the grating. Furthermore, another slit located at the non-spectrometer end of a tube ensured that only quasi-collimated light could pass to the grating such that the system effectively captured diffracted images of this slit, constituting a spectral analogue of a pinhole camera. The authors reported on proof of concept functionality by collecting spectra of light transmitted through human tissue, as well as fluorescence from a rhodamine 6 G solution, demonstrating favourable performance in comparison to contemporaneously obtained spectra from a commercially available spectrometer. System spectral resolutions of 5–10 nm were reported, with an applied grating of 1000 lines/mm. This dual aperture approach was also adopted by Oliviera et al. [28], who built an inexpensive medium density fibre-board (MDF) unit, using a DVD for the diffraction grating. A modularized design enabled both fluorescence and absorption based spectroscopic observations, with demonstrated applications in measurements of Fe^2+^ and Na^+^ in medicine and saline samples, respectively.

This approach has been elaborated on in a number of ways, the simplest of which involved the sampled light being coupled to the grating via a plastic fibre, with no additional lenses or mirrors [29]. This device had a build cost of only $10. In this case light from the smartphone flash was fibre coupled to a cuvette, for measurements of dye absorption in water samples. A second fibre coupled the transmitted radiation to the smartphone spectrometer, with detection with the phone camera, to generate a standalone system, with no requirement for external electrical or light sources. More complex transmission grating based smartphone spectrometers have typically been based on using a pinhole and a collimating lens to collimate the light, then a cylindrical focusing lens prior to the grating-phone assembly [30,31,32,33].

For example, in the works of Gallegos et al. [30] and Dutta et al. [31] the samples were placed between the collimating and cylindrical lenses, with illumination achieved using a broadband light source before the pinhole. In the former case the measurement concerned a label free photonic crystal biosensor, such that light from an external broadband light source was polarized prior to interaction with the sample. The spectrometer captured narrow shifts (with 0.009 nm accuracy) in the resonance reflection profile of this device, induced via adsorption of biomolecular assays e.g., in this case including a protein monolayer. In the study by Dutta et al. [31], a right-angled prism was placed within the optical path to enable evanescent field sensing of adsorbing media surrounding the prism. Here absorbance was measured for a number of dye solutions.

In Long et al. [32] samples were placed within a cuvette located between the broadband light source located before the pinhole. In this case measurements of Enzyme linked immunosorbent assays were demonstrated at biologically relevant concentrations; this work involved a protein biomarker used to identify cancers, and a food allergen. Fluorescence based biological assaying with a smartphone spectrometer was first demonstrated by Yu et al. [33], who used a green laser pointer to excite the sample, with the fluorescent radiation coupled into the pinhole via an optical fibre. A fluorescent molecular beam assay approach was adopted in order to detect specific microRNA nucleic acid sequences, and in particular to resolve single-base mutations between these.

In a slight variant to the above approaches, Wang et al. [34] placed a Lyot stop between the two lenses (both of which were spherical in this case) to remove unfocused scattered light. In this case the sample cuvette was placed prior to the first lens, and the spectrometer was used to measure the sample absorption of an external collimated white light source. A further notable feature was that the authors used a DVD section, rather than a commercially available diffraction grating for the first time in a smartphone spectrometer, to provide spectral dispersion; this reinforces the very low cost basis on which these instruments can be developed. This device was used to detect neurotoxins at medically relevant levels via two assay solutions. The same group subsequently extended smartphone spectroscopy to deliver on multichannel sensing for the first time, hence enabling simultaneous biosensing measurements of multiple microplates, in this case eight side-by-side samples [35]. This was achieved with the use of a microprism array, which enlarged the field of view of the smartphone sensor to match that of the underlying samples, in addition to a micro-aperture array, which mitigated against spectral crosstalk between the channels. With a total build cost of $150 this unit was used to quantify protein concentrations as well as to immunoassay a human cancer biomarker.

Another key reported approach to smartphone spectroscopy using a transmissive diffractive element is based on a so-called G-Fresnel device, placed before the camera lens. The G-Fresnel is an highly novel optical component, with a front face in the form of a Fresnel lens, and the back face rendered as a diffraction grating; this unit therefore serves to both focus and disperse incident light [36]. A key advantage of this single optical element approach is the capacity to develop very compact spectrometer units. The authors report a configuration in which radiation transmitted through a sample cuvette is coupled to the spectrometer entrance slit via an optical fibre. Using this device, the authors also demonstrated a smartphone based Bradford assay, a colourimetric analytic approach relevant to disease diagnosis and biomedical research. This procedure is based on capturing the red shifting of reagent solution absorption spectra due to binding of proteins. Here, proof of concept was demonstrated in respect of concentration measurements of bovine serum albumin, a protein derived from cows.

The final ‘transmissive grating’ smartphone spectrometer covered here incorporates polarization into the measurement protocol. In this case light enters the instrument via a slit and passes through polarization modulation optics, consisting of quarter and multiple-order wave plates, a polarizer and a collimating lens, located within a plastic case, which clips onto iPhones [37]. This protocol introduces a sinusoidal modulation in the captured spectrum, the amplitude of which is proportional to the degree to which the incident light is linearly polarized. As aerosol particles in the atmosphere act to polarize sunlight, this spectropolarimetric method can be used to characterise aerosol optical thickness, which was achieved through measurements at 550 nm in the Netherlands, via a 3000 participant strong citizen science experiment, termed the iSPEX project.

## 3. Reflection Grating Configurations

**Figure 4:**
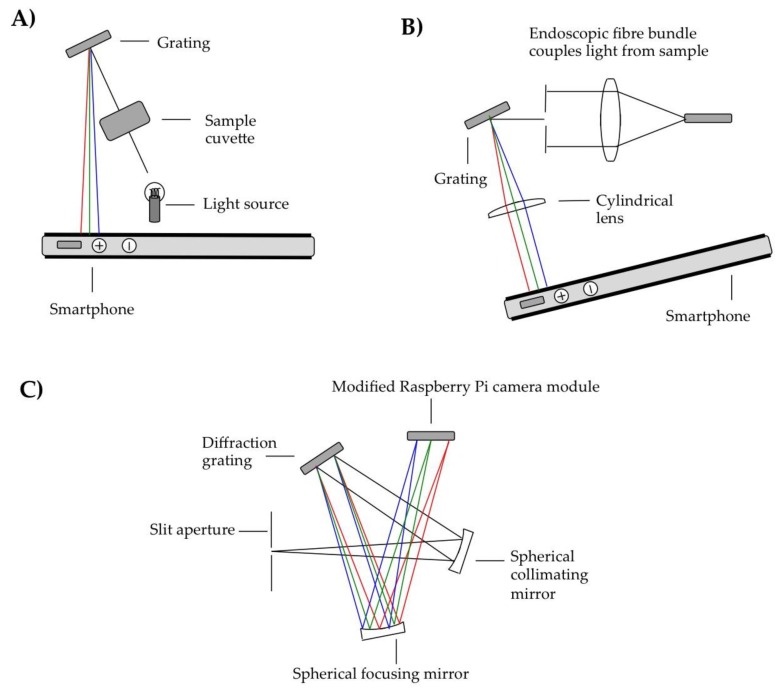
Sample developed reflective grating smartphone spectrometer concepts: (**A**) a straightforward protocol applied in both fluorescence and absorption modalities with diffraction achieved using either a CD or in-house constructed diffraction grating [38,39]; (**B**) an endoscopic approach, whereby probe type sampling was demonstrated, for example in food quality determination [40]; finally (**C**) a 3D printed Czerny turner design, incorporating a modified Raspberry Pi camera as the detector, which is based on a smartphone sensor [41]. The ray colouration provides a sense of the dispersion within these units, whereby the red rays signify the longer wavelengths, and the blue rays, the shorter ones. Please see main text for further details.

The other key modality adopted in terms of smartphone spectrometer design has been based on using reflective diffraction gratings. Sample configurations from literature reports appear in Figure 4. In the simplest case the camera flash was used as the light source, and shone through a pinhole to illuminate an absorbance sample in the optical path [38]. A compact disk was then used to disperse the light and the camera lens to image the spectrum onto the smartphone detector. Other than the smartphone camera lens, there were no further optical components, e.g., apertures or additional lenses within the optical path. Colourimetric assaying was demonstrated, in particular by detecting glucose, as well as troponin I, which is a cardiac disease biomarker in humans, using a localized surface plasmon resonance approach based on peptide functionalized gold nanoparticles.

In an earlier variant of this approach, a dual optical source configuration was deployed such that illumination could be achieved with either the camera flash or orthogonally located 370 nm UV or 450 nm blue LED sources [39]. Here, a gold-coated nano-imprinted diffraction grating was applied, instead of the CD. In this device both absorption and fluorescence spectra acquisition were demonstrated in order to probe for pH and Zn^2+^ in water samples: in the former case on the basis of a blue-absorbing, green-emitting pH sensitive fluorescent probe, and in the latter, via a UV absorbing Zn^2+^ sensitive fluoro-ionophore. An elegant design protocol was adopted, in housing all components within a single 3D printed casing, and using the smartphone battery for all the system power requirements.

The same authors have also pioneered an endoscopic fibre based reflective grating spectrometer, to enable sampling beyond the housing of the instrument assembly [40]. Such an approach could be of great utility where probe type investigation is required, for instance in agricultural produce analysis, and this furthermore eliminates potential issues caused by variation in natural illumination sources, which could otherwise be used in field measurements. In this configuration the target was illuminated via the endoscope, using the camera’s LED light. The back reflected radiation was then fibre coupled to the spectrometer, with collimation of the fibre output, followed by spatial filtering with a slit aperture, dispersion from a grating (in this case a segment from a DVD), then collimation in one plane with a cylindrical lens, before the spectral measurement with the smartphone camera. In this case spectral linewidths as low as 2 nm are reported, which to the best of our knowledge was the lowest reported up to that time from a grating based smartphone spectrometer. Whilst rather lower per pixel resolutions are quoted in a number of other articles in this domain, that parameter does not represent the true spectral resolving power, which must be characterised by measuring spectral line-widths from narrowband sources, as was indeed achieved in the case of Hossain et al. [40]. The authors of this report furthermore demonstrated proof of concept of this configuration for monitoring food quality, via measuring the absorption by organic pigments in an apple, e.g., anthocyanins, carotenoid and chlorophyll.

To date, smartphone spectroscopy has been restricted to the visible spectral region, owing to the rather non-UV-transmissive optical configurations normally applied in these cameras, e.g., the Bayer filters typically applied to the fore of the sensors in order to generate RGB mosaics. To broaden the spectral coverage of this approach, the Bayer layer has recently been chemically removed [24] from sensors designed for the smartphone market, but instead located in inexpensive Raspberry Pi camera boards, which are controlled by Raspberry Pi computers. Such a procedure is clearly far easier to implement on a sensor located outside, rather than within the housing of a smartphone, in order to deliver on usable UV sensitivity.

**Figure 5:**
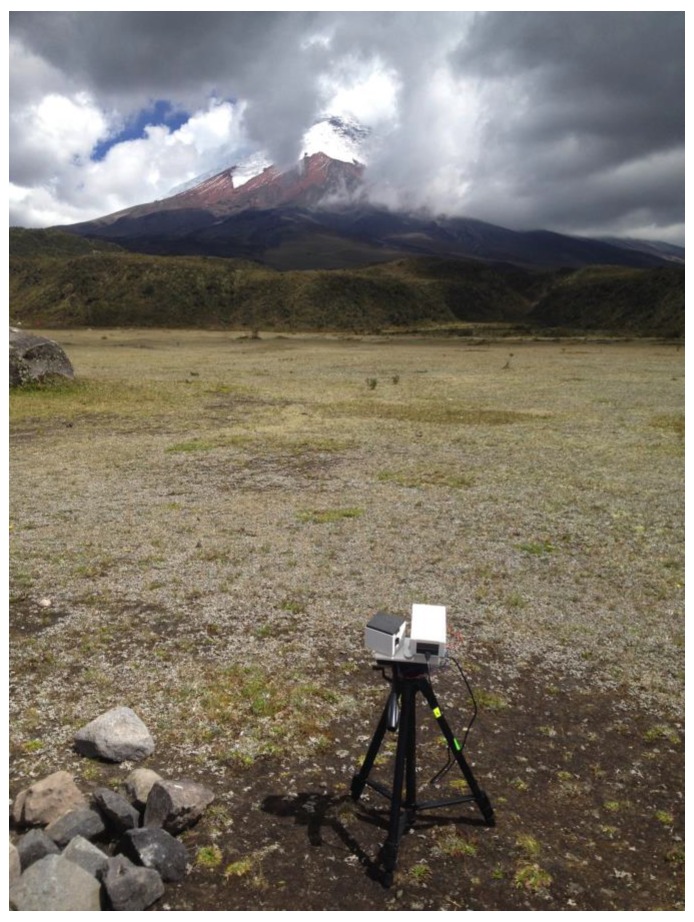
Raspberry Pi camera-based spectrometer applied to measurements of sulphur dioxide gas release from Cotopaxi volcano, Ecuador.

This sensor arrangement has furthermore been implemented in a detector capacity within a compact 3D printed Czerny-Turner spectrometer design, to deliver on 1 nm resolution spanning an ultraviolet spectral range, to constitute the narrowest linewidth reported yet from a grating based smartphone sensor based spectrometer [41]. This device has been used to perform absorption-based measurements at ≈310 nm of sulphur dioxide gas in the atmosphere, e.g., concerning gas release from volcanoes (Figure 5), where the emissions of this species are routinely captured in volcano monitoring operations, and a low cost solution could be highly useful given that most hazardous volcanoes are located in less economically developed countries [42,43]. This, with the earlier iSPEX unit aerosol measurements [37], move smartphone spectroscopy beyond being a tool for in-situ analysis, by including a remote sensing modality in addition.

## 4. Discussion

The sections above draw attention to the various applied approaches to capturing spectra with smartphone cameras, based on reflective and transmissive grating configurations, the dominant approaches applied hitherto in the field of smartphone spectroscopy; we also discuss the application of these devices in a number of scientific fields. In terms of these two approaches, there has yet to be a clear convergence on either modality. Transmissive gratings provide the benefit of low polarization sensitivity, as well as the possibility of ultra compact in-line operation, but also carry the potential for greater absorption losses. This said, folded spectrometer designs with reflective gratings can been applied in order to generate devices of usable resolution in small footprint packages [41].

Gratings of either format are available at low cost as illustrated above, e.g., DVDs have been applied in some of the reflective grating units. There is also the choice of blazed or holographic gratings. Whilst the former can provide higher efficiencies close to the blaze angle, therefore suitability for low light applications, there are greater issues with ghosting. In contrast, stray light is minimized with holographic devices, which also permit higher groove densities, hence suitability for higher spectral resolution applications. For in depth reviews of the applications of smartphones in key target domains, where a wider variety of sensing modalities are applied, we refer the reader to alternate articles, e.g., [44] in respect of food monitoring and [2] concerning microscopy.

The smartphone spectrometers covered here range from very simple units based on two slits [27], to more complex arrangements involving polarization [37] as well as the rather complex G-Fresnel element—a hybrid component, which acts as both a lens and diffraction grating [36]. Grating based smartphone spectroscopy has even been realised by coupling very low cost educational spectroscopy kits to smartphones, where card based spectrometers provide dispersion of incident radiation [45]. Such devices have been applied in metabolomics applications, e.g., in terms of drug toxicity monitoring [46]. Hence, smartphone spectroscopy now offers the possibility to capture and analyse spectra at an unprecedented price point, with quoted build costs as low as $10 [29], based on the minimal associated materials costs, e.g., by using DVDs for the diffraction grating e.g., [28], and fabricating the device housings from readily available household materials [27], e.g., MDF [28], or more commonly with 3D printing e.g., [39,41]. The latter technology, in particular, with its capacity to manufacture highly customizable optical hardware, has been a major reason for the recent expansion in user-built smartphone spectrometers [26].

There has also been some work focused on alternate smartphone spectrometer architectures: for instance, on coupling the smartphones to external compact ‘micro-spectrometers’, which contain their own detector units, for example a wirelessly connected, ultracompact grating based device of 15 nm spectral resolution, which was applied for non-destructive monitoring of fruit ripeness [47]. Fruit monitoring has also been achieved with another hand held compact spectrometer, based on a linear variable filter, suitable for Bluetooth networking with a smartphone [48]. Indeed, the application of micro-spectrometers shows considerable future promise in smartphone spectroscopy, whether or not the phone camera is applied as the sensor. These MEMS (microelectromechanical system) architectures involve both grating and non-grating, e.g., interferometeric [49] configurations, in the latter case avoiding the disadvantage of spectral resolution scaling with unit size in the former scenario [50]. There have been a number of significant recent advances in the micro-spectrometer field, which may well prove relevant to smartphone sensing, e.g., based on colloidal quantum dot [51], linear-variable optical filter [52], integrated filter array [53] and disordered photonic chip [54] arrangements, which are highly suitable for lab on a chip applications.

Another key class of smartphone spectrometers concerns non-grating based devices, which make use of the phone camera as the device sensor. Although far less ubiquitous than the grating approaches detailed in the prior two sections, this modality has received some attention within the field of smartphone spectroscopy and potential routes to realisation of such architectures are detailed below. This generic class of spectrometer relies on other approaches to wavelength division, e.g., optical band-pass filtering. To this end, in the most basic sense, any colour camera could be used as a three-band spectrometer, such that the channels provide a measurement of the relative brightness of red, green and blue within the scene. The logical extension to this approach is adoption of linear variable filters, as has been applied within the micro-spectrometer architectures detailed above [52]. In this case, the spectra generated by such filters replace those projected onto the sensor focal plane array by gratings, enabling spectral acquisition in highly compact units, by avoiding the potentially rather large internal path length requirements of grating based devices. Such filters at present provide wavelength resolution, which is roughly an order of magnitude lower than that of grating approaches, with filter costs approximately an order of magnitude greater than that of gratings. Hence, whilst there is undoubtedly considerable potential for these units to be coupled to smartphone cameras, price reduction is a key factor for future applicability. Other potential approaches could involve sweeping the illumination wavelength of the scene or object; this could be achieved with swept-source lasers or acousto-optic tunable filters. The latter devices are based on piezo-electric transducers bonded to typically anisotropic media, within which the refractive index is periodically redistributed, in order to enable spectral tunability of the transmitted radiation. This approach also holds promise in that devices can be developed with rather compact form factors. Similarly, chiral (twisted helix) liquid crystals could be applied here, such that the reflection spectra of these units is adjustable, according to the pitch of the helix, which can be controlled via application of an electric field. In principle, this approach could also be developed in a miniature format, providing a rather wider bandwidth than from diffraction gratings, however this would also require precise temperature stabilisation. One non-grating based approach which has gained traction is the Fringoe device [55]. This unit is based on an array of Mach Zehnder interferometers, which generate an interferogram across the sensor array, which is then subjected to a Fourier Transform to output spectra. Hence this unit effectively operates as a Fourier Transform spectrometer, but without the requirement for mechanical scanning, and with an exceptionally small footprint and quoted spectral resolution of a nanometer or better.

To date smartphone spectrometers have been applied in a number of measurement configurations, with foci on in-situ absorption e.g., [31,32,34,39], fluorescence [33,39,41] and photonic crystal based detection [30]. In terms of application areas, this work has been focused on: biomedical research, food science and environmental monitoring e.g., [33,34,35,36,37]. However, as identified by the articles cited in this paper, smartphone spectrometers also hold promise in a far wider variety of domains, for example, lifestyle monitoring, home diagnostics [38], forensic spectroscopy [40], education [27], as well as with respect to any liquid assay approach based on light emission, e.g., including chemiluminescence and phosphorescence, in addition to the already demonstrated fluorescence measurements. A particular new frontier concerns open path atmospheric spectroscopy, based on demonstrations of smartphone sensor based measurements of sulphur dioxide in volcanic plumes [41] and spectropolarimetric aerosol optical thickness measurements [37]. This approach could readily be extended to other spectral regions e.g., to monitor water vapour and ozone concentrations, in addition. At this point capture of UV spectra with these sensors has been based on sensors located outside of the smartphone body. However, as some manufacturers use UV transmissive optics (e.g., a sapphire lens on the iPhone) and others, monochrome sensors (e.g., on some Huawei units), on-phone capture of UV spectra may become a reality going forward. A key step in this direction has been achieved by pointing cameras directly at the sun and capturing UVA solar irradiances and aerosol optical depths [56]. Capture of UV spectra, could be of use for weighting with the relevant action spectrum to determine the biologically effective UV for a variety of processes, e.g., in terms of the previtamin D_3_ synthesis action spectrum (up to 330 nm) and the erythema action spectrum [CIE, 1998]. As has already been illustrated with the iSPEX project, this sort of hardware has great promise for widespread proliferation to broaden current monitoring data, particularly in concert with the citizen science community [37].

The current literature on smartphone spectrometers clearly alludes to the benefits of these units, namely: the possibility to straightforwardly reconfigure devices in terms of their resolution, spectral range and coverage e.g., [27], for example by changing slits, and/or transferring the internal optics between 3D printed housings, each ‘tuned’ for a different application area. There is also the small footprint of these units e.g., [35,36], which are a fraction of the sizes of the laboratory instrumentation conventionally applied in many of the discussed application areas, in addition to low unit build costs, which could be further reduced if manufacturing were to be performed at scale [36]. A further related factor is the real field portability of these devices, as well as the scope for the entire measurements system e.g., including light sources, to be powered from the phone battery and controlled via the phone software [39]. All of this has the potential to revolutionise in-field diagnostics, which could be of major significance for point of care medical applications, particularly in resource poor regions. This carries particular advantages, for instance in terms of biochemical analyses of perishable samples, e.g., protein assays [36], especially given that multi-channel operation has been demonstrated, to enable high throughput functionality [35].

A further key to the potential applicability of these units, particularly in the medical domain, is the phone processing capacity, a point made by Kwon et al. [46], e.g., in terms of delivering streamlined omics analyses and data visualization. Finally, a number of authors point to the benefits of acquiring spectra, rather than just RGB output or single/few bandpass filter data. For example, Long et al. [32] note that in their application area, access to continuous spectra provides scope to reduce measurement detection limits considerably, relative to single wavelength operation. In addition, Yu et al. [33] note the benefit this brings in terms of being able to better filter out noise sources, which are at wavelengths other than the signal, e.g., scattered excitation source radiation for fluorescence observations.

With these benefits in mind, a number of authors have performed comparisons against laboratory instrumentation applied in these application areas, with generally positive outcomes, e.g., Oliviera et al. [28] note no statistically significant differences in absorption and emission measurements with their device vs. commercial spectrometers. Similarly, Long et al. [32] compared their smartphone sensor absorption based enzyme assay approach to a conventional microplate reader, demonstrating detection limits, which were comparable or superior to those of the latter unit. Indeed, comparable/better performance with respect to a number of commercial devices is also alluded to in a number of other articles [33,35], in particular pointing towards the smartphone spectrometers having sufficient sensitivity to be usable in a wide variety of application areas [33], and satisfying criteria required in clinical applications [35]. 

There is obviously the caveat that these units cannot be expected to operate at the level of all conventionally applied commercial technology [33,35], given that the smartphone spectrometers are rather, and in some cases vastly, cheaper devices. However, given the very low smartphone spectrometer build pricing, the relative performance is impressive indeed. There are of course also additional steps that could be taken in attempts to improve the smartphone spectrometer signal to noise characteristics, for instance, the 2D sensor allows for co-adding in one dimension, the spectra acquired across the other plane of the sensor [41]. A final consideration here is the thermal and mechanical stability of the units, in terms of the largely 3D printed architectures that have been adopted here. Further study is required in order to ascertain how this varies relative to pre-existing commercially distributed spectrometer units.

The key distinguishing factors between the laboratory and smartphone spectrometer units are cost and physical size. Whilst smartphone devices of course carry the potential advantages of minimising both of these parameters, there are still fundamental aspects of grating based spectrometer design which cannot be overcome, e.g., a reduction in spectral resolution with shrinking of the bench size. Reduced cost could also limit the quality of internal optics, with a potentially adverse bearing upon spectral absorbing power, and there is furthermore the issue of the compact sensor sizes, such that typical commercially available spectrometer detectors would involve wider linear arrays to expedite capture of wider spectral ranges. This said, we have recently achieved acquisition of nanometer resolution spectra, to enable atmospheric spectroscopy applications, from a smartphone sensor based unit costing only a few hundred dollars. Therefore, it does appear, that with judicious choices of optical configuration that at least relatively high performance operation is readily achievable [41]. In terms of the desired combination of low cost and compactness MEMS type architectures are likely to play an increasing role going forward. One key technology in this regard, which has the potential for low cost mass production is the G-Fresnel device, which has both nanometer quoted spectral resolution and minimal dimensions (1.8′′ × 0.8′′ × 0.9′′) [33].

## 5. Conclusions

Here we have covered the exciting recent development of smartphone spectrometer science, including developed hardware architectures and demonstrated application areas. Given that these units have displayed impressive performance with respect to hitherto applied laboratory instrumentation, and that the devices are inexpensive and highly field portable, they have the potential to revolutionise fields such as medical diagnostics, environmental monitoring and food quality inspection in the coming years. This follows a wider technological trend of cost reduction and miniaturization in instrumentation, for instance, ‘Mini-ion’ devices for gene sequencing, which are having highly disruptive impacts in field science, by delivering high throughput data even in the most extreme, e.g., glacial field environments [57]. Another key arena has been that of drone/UAV technology, which from early studies e.g., [58] has led to ubiquitous impacts on a number of scientific arenas e.g., [59], driven by miniaturization and cost reduction in the applied units. 

In terms of future outlook a key frontier will be dissemination of these units in a more wide scale capacity than has been achieved hitherto, following the pioneering example of the iSPEX citizen science project [37]. Indeed, there is now increasing evidence of smartphone spectrometer hardware spreading beyond the maker and academic research communities and into the commercial sector, in view of the significant available market opportunities, which build on the already ubiquitous smartphone technology. To this end there are now a number of ventures, at various stages in their business development plans, focused on bringing smartphone spectrometers to the market place, e.g., [55,60,61,62,63,64]. At present this activity is focused on discrete add on devices, which are developed by third parties and can be optionally fitted to the smartphones, and as yet there appears not to be convergence on the adopted spectra generation modality. However, going forward it may be that the smartphone manufacturers themselves elect to develop built-in dedicated spectral channels within the assembly of the phones themselves. This could be achieved by removing the camera sensor Bayer filters and replacing with linear variable filters or adopting compact MEMS architectures to deliver on ultra-compact form factors. Embedded smartphone spectrometers could open up a considerable array of security and data encoding applications, e.g., advanced spectral QR codes, and personal monitoring of food quality in store or at home in order to reduce waste and prevent food poisoning.

Going forward, another key issue will be the integrity of the spectra acquired with the smartphones. To date some of the reports detailed above are likely to have been affected by automatic adjustments in the camera acquisition settings, for example gain and exposure time, which play a valuable role in camera image capture, but which will pose problems where quantitative data acquisition and processing is required. It is essential that full software control of these parameters is applied going forward, therefore. In terms of software, the processing capacity of the phone itself may also play an increasing role in data analysis, following on from recently demonstrated metabolomics analyses of acquired spectra from smartphones [42,43]. Another factor is the ongoing improvements of the camera sensors themselves, in particular with the trickle down benefits of progress in CMOS devices e.g., for the digital SLR photography market and especially in terms of the pixel density. As smartphone sensors have been demonstrated to perform at least adequately for a number of application areas, and in a number of cases as well as rather more expensive commercial units, this advancement in sensor technology should prove to be highly enabling for the quality of data capture and the potential applicability of smartphone spectrometers going forward [28,32,33,35,41]. Finally, there is likely to be increased usage of both dimensions of the smartphone sensor, either through co-adding of spectra in software over the non-spectral axis of the array of the array in order to increase signal to noise [41] or including image acquisition in the measurement [27,65]. Indeed, on the basis of all the above benefits and potential future directions, as well as the advent of the Internet of Things and cloud computing it appears as though smartphone spectrometers may now be in the verge of becoming highly ubiquitous devices, capable of democratizing spectroscopy to a completely unprecedented degree.

## References

[1] Breslauer D.N., Maamari R.N., Switz N.A., Lam W.A., Fletcher D.A. (2009). Mobile phone based clinical microscopy for global health applications. PLoS ONE.

[2] Contreras-Naranjo J.C., Wei Q., Ozcan A. (2016). Mobile phone-based microscopy, sensing and diagnostics. IEEE J. Sel. Top. Quantum Electron..

[3] Bastawrous A., Rono H.K., Livingstone I.A.T., Weiss H.A., Jordan S., Kuper H., Burton M.J. (2015). Development and Validation of a Smartphone-Based Visual Acuity Test (Peek Acuity) for Clinical Practice and Community-Based Fieldwork. JAMA Opthamol..

[4] Bastawrous A., Giardini M.E., Bolster N.M., Peto T., Shah N., Livingstone I.A.T., Weiss H.A., Hu S., Rono H., Kuper H. (2016). Clinical validation of a smartphone-based adapter for optic disc imaging in Kenya. JAMA Opthamol..

[5] Sumriddetchkajorn S., Chaitavon K., Intaravanne Y. (2014). Mobile-phone based colorimeter for monitoring chlorine concentrations in water. Sens. Actuator B Chem..

[6] Sicard C., Glen C., Aubie B., Wallace D., Jahanshahi-Anhuhi S., Pennings K., Daigger G.T., Pelton R., Brennan J.D., Felipe C.D.M. (2015). Tools for water quality monitoring and mapping using paper-based sensors and cell phones. Water Res..

[7] Vashist S.K., van Oordt T., Schneider E.M., Zengerle R., von Stetten F., Luong J.H.T. (2015). A smartphone-based colorimeter reader for bioanalytical applications using the screen-based bottom illumination provided by gadgets. Biosens. Bioelectron..

[8] Yang X., Wang Y., Liu W., Zhang Y., Zheng F., Wang S., Zhang D., Wang J. (2016). A portable system for on-site quantification of formaldehyde in air based on G-quadruplex halves coupled with a smartphone reader. Biosens. Bioelectron..

[9] Gómez-Robledo L., López-Ruiz N., Melgosa M., Palma A.J., Capitán-Vallvey L.F., Sánchez-Marañón M. (2013). Using the mobile phone as Munsell soil-colour sensor: An experiment under controlled illumination conditions. Comput. Electron. Agric..

[10] Canning J., Lau A., Naqshbandi M., Petermann I., Crossley M.J. (2011). Measurement of Fluorescence in a Rhodamine-123 Doped Self-Assembled “Giant” Mesostructured Silica Sphere Using a Smartphone as Optical Hardware. Sensors.

[11] Hossain M.A., Canning J., Yu Z., Ast S., Rutledge P., Wong J.K.-H., Jamalipour A., Crossley M.J. (2017). Time-resolved and temperature tuneable measurements of fluorescent intensity using a smartphone fluorimeter. Analyst.

[12] Zhang C., Kim J.P., Creer M., Yang J., Liu Z. (2017). A smartphone-based chloridometer for point-of-care diagnostics of cystic fibrosis. Biosens. Bioelectron..

[13] Ghassemi P., Wang B., Wang J., Wang Q., Chen Y., Pfefer T.J. (2017). Evaluation of Mobile Phone Performance for Near-Infrared Fluorescence Imaging. IEEE Trans. Biomed. Eng..

[14] Debus B., Kirsanov D., Yaroshenko I., Legin A. (2017). A simple design atomic emission spectrometer combined with multivariate image analysis for the determination of sodium content in urine. Anal. Methods.

[15] Hossain M.A., Canning J., Cook K., Jamalipour A. (2015). Smartphone laser beam spatial profiler. Opt. Lett..

[16] Hassannejad H., Matrella G., Ciampolini P., De Munari I., Mordonini M., Cagnoni S. (2017). A new approach to image-based estimation of food volume. Algorithms.

[17] Anderson K., Griffiths D., DeBell L., Hancock S., Duffy J.P., Shutler J.D., Reinhardt W.J., Griffiths A. (2016). A grassroots remote sensing toolkit using live coding, smartphones, kites and lightweight drones. PLoS ONE.

[18] Campos-Taberner M., García-Haro F.J., Confalonieri R., Moreno A., Sánchez-Ruiz S., Gilabert M.A., Camachi F., Boschetti M., Busetto L. (2016). Multitemporal Monitoring of Plant Area Index in the Valencia Rice District with PocketLAI. Remote Sens..

[19] Parisi A.V., Downs N., Igoe D., Turner J. (2016). Characterisation of cloud cover with a smartphone camera. Instrum. Sci. Technol..

[20] Igoe D., Parisi A.V. (2015). Evaluation of a Smartphone Sensor to Broadband and Narrowband Ultraviolet A Radiation. Instrum. Sci. Technol..

[21] Turner J., Parisi A.V., Igoe D.P., Amar A. (2017). Detection of ultraviolet B radiation with internal smartphone sensors. Instrum. Sci. Technol..

[22] Igoe D.P., Amar A., Parisi A.V., Turner J. (2017). Characterisation of a smartphone image sensor response to direct solar 305 nm irradiation at high air masses. Sci. Total Environ..

[23] Igoe D., Parisi A.V., Carter B. (2013). Characterisation of the UVA response of a smart phone. Photochem. Photobiol..

[24] Wilkes T.C., McGonigle A.J.S., Pering T.D., Taggart A.J., White B.S., Bryant R.G., Willmott J.R. (2016). Ultraviolet Imaging with Low Cost Smartphone Sensors: Development and Application of a Raspberry Pi-Based UV Camera. Sensors.

[25] Wilkes T.C., Pering T.D., McGonigle A.J.S., Tamburello G., Willmott J.R. (2017). A Low-Cost Smartphone Sensor-Based UV Camera for Volcanic SO_2_ Emission Measurements. Remote Sens..

[26] Zhang C., Anzalone N.C., Faria R.P., Pearce J.M. (2013). Open-source 3D-printable optics equipment. PLoS ONE.

[27] Smith Z.J., Chu K., Espenson A.R., Rahimzadeh M., Gryshuk A., Molinaro M., Dwyre D.M., Lane S., Matthews D., Wachsmann-Hogiu S. (2011). Cell-phone-based platform for biomedical device development and educational applications. PLoS ONE.

[28] De Oliveira H.J.S., de Almeida P.L., Sampaio B.A., Fernandes J.P.A., Pessoa-Neto O.D., de Lima E.A., de Almeida L.F. (2017). A handheld smartphone-controlled spectrophotometer based on hue to wavelength conversion for molecular absorption and emission measurements. Sens. Actuators B Chem..

[29] Özdemir G.K., Bayram A., Kiliç V., Horzum N., Solmaz M.E. (2017). Smartphone-based detection of dyes in water for environmental sustainability. Anal. Methods.

[30] Gallegos D., Long K.D., Yu H., Clark P.P., Lin Y., George S., Nath S., Cunningham B.T. (2013). Label-free biodetection using a smartphone. Lab Chip.

[31] Dutta S., Choudhury A., Nath P. (2014). Evanescent wave coupled spectroscopic sensing using smartphone. IEEE Photon. Technol. Lett..

[32] Long K.D., Yu H., Cunningham B.T. (2014). Smartphone instrument for portable enzyme-linked immunosorbent assays. Biomed. Opt. Express.

[33] Yu H., Tan Y., Cunningham B.T. (2014). Smartphone fluorescence spectroscopy. Anal. Chem..

[34] Wang L.-J., Chang Y.-C., Ge X., Osmanson A.T., Du D., Lin Y., Lei L. (2016). Smartphone optisensing platform using a DVD grating to detect neurotoxins. ACS Sens..

[35] Wang L.-J., Chang Y.-C., Sun R., Li L. (2017). A multichannel smartphone optical biosensor for high-throughput point-of-care diagnostics. Biosens. Bioelectron..

[36] Zhang C., Cheng G., Edwards P., Zhou M.-D., Zheng S., Liu Z. (2016). G-Fresnel smartphone spectrometer. Lab Chip.

[37] Snik F., Rietjens J.H.H., Apituley A., Volten H., Majling B., Di Noia A., Heikamp S., Heinsbroek R.C., Hasekamp O.P., Smit J.M. (2014). Mapping atmospheric aerosols with a citizen science network of smartphone spectropolarimeters. Geophys. Res. Lett..

[38] Wang Y., Liu X., Chen P., Tran N.T., Zhang J., Chia W.S., Boujday S., Liedberg B. (2016). Smartphone spectrometer for colorimetric biosensing. Analyst.

[39] Hossain M.A., Canning J., Ast S., Cook K., Rutledge P.J., Jamalipour A. (2015). Combined “dual” absorption and fluorescence smartphone spectrometers. Opt. Lett..

[40] Hossain M.A., Canning J., Cook K., Jamalipour A. (2016). Optical fibre smartphone spectrometer. Opt. Lett..

[41] Wilkes T.C., McGonigle A.J.S., Willmott J.R., Pering T.D., Cook J.M. (2017). Low-cost 3D printed 1 nm resolution smartphone sensor-based spectrometer: Instrument design and application in ultraviolet spectroscopy. Opt. Lett..

[42] Kantzas E.P., McGonigle A.J.S. (2008). Ground based ultraviolet remote sensing of volcanic gas plumes. Sensors.

[43] Galle B., Oppenheimer C., Geyer A., McGonigle A.J.S., Edmonds M., Horrocks L. (2002). A miniaturized ultraviolet spectrometer for remote sensing of SO_2_ fluxes: A new tool for volcano surveillance. J. Volcanol. Geotherm. Res..

[44] Rateni G., Dario P., Cavallo F. (2017). Smartphone based food diagnostic technologies: A review. Sensors.

[45] Kwon H., Park J., An Y., Sim J., Park S. (2014). A smartphone metabolomics platform and its application to the assessment of cisplatin-induced kidney toxicity. Anal. Chim. Acta.

[46] Kwon H.N., Phan H.-D., Xu W.J., Ko Y.-J., Park S. (2016). Application of a smartphone metabolomics platform to the authentication of *Schisandra sensesis*. Phytochem. Anal..

[47] Das A.J., Wahi A., Kothari I., Raskar R. (2016). Ultra-portable, wireless smartphone spectrometer for rapid, non-destructive testing of fruit ripeness ripeness. Sci. Rep..

[48] Yu X., Lu Q., Gao H., Ding H. (2016). Development of a handheld spectrometer based on a linear variable filter and a complementary metal-oxide-semiconductor detector for measuring the internal quality of fruit. J. Near Infrared Spectrosc..

[49] Knipp D., Stiebig H., Bhalotra S.R., Bunte E., Kung H.L., Miller D.A.B. (2005). Silicon based micro-Fourier spectrometer. IEEE Trans. Electron Devices.

[50] Wolffenbuttel R.F. (2005). MEMS-based optical mini- and microspectrometers for the visible and infrared spectral range. J. Micromech. Microeng..

[51] Bao J., Bawendi M.G. (2015). A colloidal quantum dot spectrometer. Nature.

[52] Emadi A., Wu H., de Graaf G., Wolffenbuttel R. (2012). Design and implementation of a sub-nm resolution microspectrometer based on a linear-variable Optical Filter. Opt. Express.

[53] Wang S.-W., Xia C., Chen X., Lu W., Li M., Wang H., Zheng W., Zhang T. (2007). Concept of a high-resolution miniature spectrometer using an integrated filter array. Opt. Lett..

[54] Redding B., Liew S.F., Sarma R., Cao H. (2013). Compact spectrometer based on a disordered photonic chip. Nat. Photon..

[55] FrinGOe. https://fringoe.com.

[56] Igoe D.P., Parisi A., Carter B. (2014). Smartphone-based android app for determining UVA aerosol optical depth and direct solar irradiances. Photochem. Photobiol..

[57] Edwards A., Debbonaire A.R., Sattler B., Mur L.A.J., Hodson A.J. (2016). Extreme metagenomics using nanopore DNA sequencing: A field report from Svalbard, 78 N. bioRxiv.

[58] McGonigle A.J.S., Aiuppa A., Giudice G., Tamburello G., Hodson A.J., Gurrieri S. (2008). Unmanned aerial vehicle measurements of volcanic carbon dioxide fluxes. Geophys. Res. Lett..

[59] Villa T.F., Gonzalez F., Miljievic B., Ristovski Z.D., Morawska L. (2016). An Overview of Small Unmanned Aerial Vehicles for Air Quality Measurements: Present Applications and Future Prospectives. Sensors.

[60] Lighting Passport. https://www.lightingpassport.com.

[61] Scio-Phone. https://www.phone.consumerphysics.com.

[62] Eigen Imaging. http://www.eigenimaging.com/smartphone-spectrometer.

[63] Stratio Technology. http://www.stratiotechnology.com.

[64] Public Lab. https://publiclab.org/wiki/smartphone-spectrometer.

[65] Cai F., Wang D., Zhu M., He S. (2017). Pencil-line imaging spectrometer for bio-samples sensing. Biomed. Opt. Express.

